# Replacement of Glycoprotein B in Alcelaphine Herpesvirus 1 by Its Ovine Herpesvirus 2 Homolog : Implications in Vaccine Development for Sheep-Associated Malignant Catarrhal Fever

**DOI:** 10.1128/mSphere.00108-16

**Published:** 2016-08-03

**Authors:** Cristina W. Cunha, Naomi S. Taus, Benjamin G. Dewals, Alain Vanderplasschen, Donald P. Knowles, Hong Li

**Affiliations:** a Animal Disease Research Unit, Agricultural Research Service, USDA, Pullman, Washington, USA; b Department of Veterinary Microbiology and Pathology, Washington State University, Pullman, Washington, USA; c Fundamental and Applied Research in Animals and Health (FARAH), Immunology-Vaccinology, Faculty of Veterinary Medicine, University of Liège, Liège, Belgium; UNC-Chapel Hill

**Keywords:** Chimeric virus, alcelaphine herpesvirus 1, malignant catarrhal fever, ovine herpesvirus 2, vaccine

## Abstract

Vaccine development is a top priority in malignant catarrhal fever (MCF) research. In the case of sheep-associated MCF (SA-MCF) caused by ovine herpesvirus 2 (OvHV-2), progress toward this objective has been hindered by the absence of methods to attenuate or modify the virus, since it cannot be propagated *in vitro*. As an alternative for vaccine development, in this study, we tested the hypothesis that one of the SA-MCF vaccine candidate targets, OvHV-2 glycoprotein B (gB), could be expressed by a nonpathogenic alcelaphine herpesvirus 1 (AlHV-1) and then evaluated the potential of the AlHV-1/OvHV-2 chimera to be used as a vaccine and a diagnostic tool. The construction and characterization of an AlHV-1/OvHV-2 chimeric virus that is nonpathogenic and expresses an OvHV-2 vaccine target are significant steps toward the development of an SA-MCF vaccine and also provide a valuable means to study OvHV-2 biology.

## Abstract (precis)

Vaccine development is a top priority in malignant catarrhal fever (MCF) research. In the case of sheep-associated MCF (SA-MCF) caused by ovine herpesvirus 2 (OvHV-2), progress toward this objective has been hindered by the absence of methods to attenuate or modify the virus, since it cannot be propagated *in vitro*. As an alternative for vaccine development, in this study, we tested the hypothesis that one of the SA-MCF vaccine candidate targets, OvHV-2 glycoprotein B (gB), could be expressed by a nonpathogenic alcelaphine herpesvirus 1 (AlHV-1) and then evaluated the potential of the AlHV-1/OvHV-2 chimera to be used as a vaccine and a diagnostic tool.

## Opinion/Hypothesis

Ovine herpesvirus 2 (OvHV-2) and alcelaphine herpesvirus 1 (AlHV-1) are two major gammaherpesviruses in the malignant catarrhal fever (MCF) group within the genus *Macavirus* (1, 2). OvHV-2 and AlHV-1 are carried asymptomatically by sheep and wildebeest, respectively, but can cause MCF when transmitted to nonadapted species. MCF is an often fatal lymphoproliferative disease that affects a large variety of animals, including cattle, bison, deer, pigs, and antelope (2, 3). Although the disease is sporadic, significant outbreaks have been reported with death of large numbers of animals. With no treatment available, separation of carrier and clinically susceptible animals is currently the only disease control strategy. Better ways to avoid virus transmission and disease are necessary, and the development of vaccines is a high priority in MCF research.

An attenuated strain of AlHV-1, obtained by successive passages in culture (4), protected cattle against lethal challenge with the virulent virus, and the protection was associated with high levels of neutralizing antibodies in nasal secretions (5, 6). AlHV-1 and OvHV-2 are very close genetically and cause clinically and pathologically indistinguishable diseases; however, using the attenuated AlHV-1 as a vaccine against OvHV-2-induced MCF is unlikely to succeed because there is no cross-reactivity of neutralizing antibodies between the two viruses (7). Moreover, because there is no *in vitro* system to culture OvHV-2, the same strategy used to attenuate AlHV-1 cannot be used with OvHV-2. A possible strategy to overcome these problems would be to modify AlHV-1, which can be propagated *in vitro*, to express protective OvHV-2 antigens. Recently, a mutated AlHV-1 (AlHV-1^ΔORF73^), which has the gene encoding the latency-associated nuclear antigen disrupted, was shown to be attenuated; AlHV-1^ΔORF73^ is able to infect and replicate in rabbits without causing disease, and it induces protection against lethal challenge with a virulent AlHV-1 (8). Using this mutant virus, we tested the overall hypothesis that AlHV-1 with its glycoprotein B (gB) gene replaced by the OvHV-2 homologous gene can replicate *in vitro* and is infectious to rabbits. OvHV-2 gB stimulates neutralizing antibodies capable of blocking OvHV-2 entry (9), and therefore, it was chosen as a target in this study. Here we describe the construction and characterization of the AlHV-1/OvHV-2 chimeric virus and indicate its potential as a vaccine and as a tool for analysis of OvHV-2 neutralizing antibody responses.

**FIG 1:**
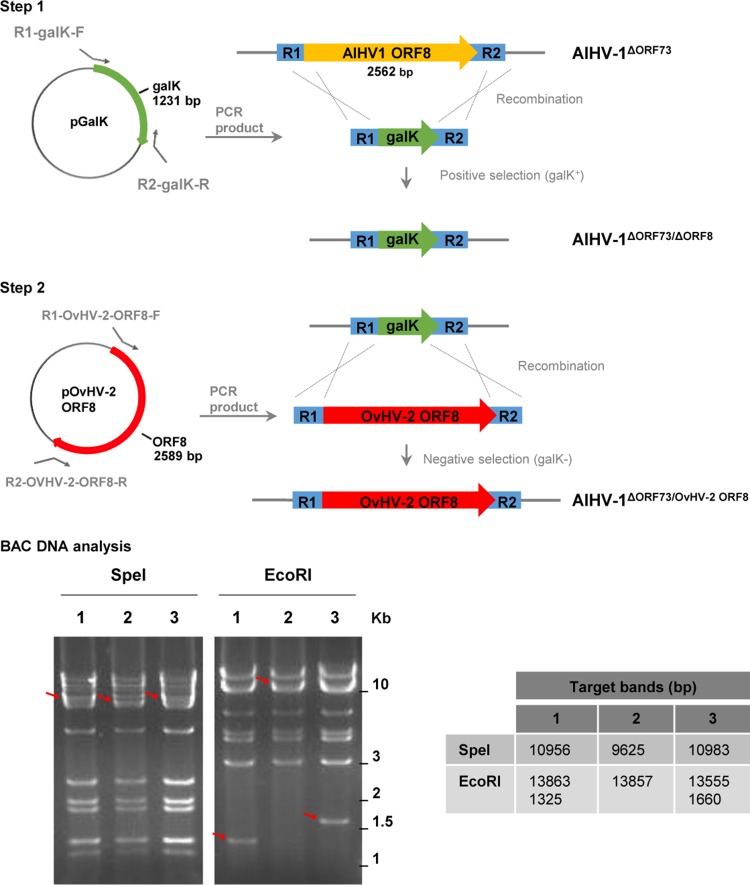
Replacement of AlHV-1 ORF8 by OvHV-2 ORF8 in the AlHV-1^ΔORF73^ BAC using the *galK* recombineering system. (Top) In step 1, the *galK* gene sequence flanked by arms (R1 and R2) corresponding to AlHV-1 ORF8 was produced by PCR and transformed into *E. coli* SW102 containing the AlHV-1^ΔORF73^ BAC. Positive selection on minimal medium containing galactose was used to identify colonies carrying the *galK* gene (AlHV-1^ΔORF73 ΔORF8^). In step 2, a clone with the AlHV-1 ORF8 replaced by *galK* was subjected to recombination with a PCR fragment containing the OvHV-2 ORF8 gene flanked by the R1 and R2 arms. Negative selection was performed on plates containing minimal medium and 2-deoxygalactose with glycerol as the carbon source and clones carrying OvHV-2 ORF8 (AlHV-1^ΔORF73/OvHV-2-ORF8^) were selected. (Bottom) Restriction patterns of the BAC DNAs are demonstrated following digestion with SpeI and EcoRI and electrophoresis in 1% agarose/SYBR green gel. Lanes: 1, AlHV-1^ΔORF73^; 2, AlHV-1^ΔORF73 ΔORF8^; 3, AlHV-1^ΔORF73/OvHV-2-ORF8^. Red arrows indicate shifts in band size or new bands. The expected sizes of target bands are indicated in base pairs to the right of the gels.

**FIG 2:**
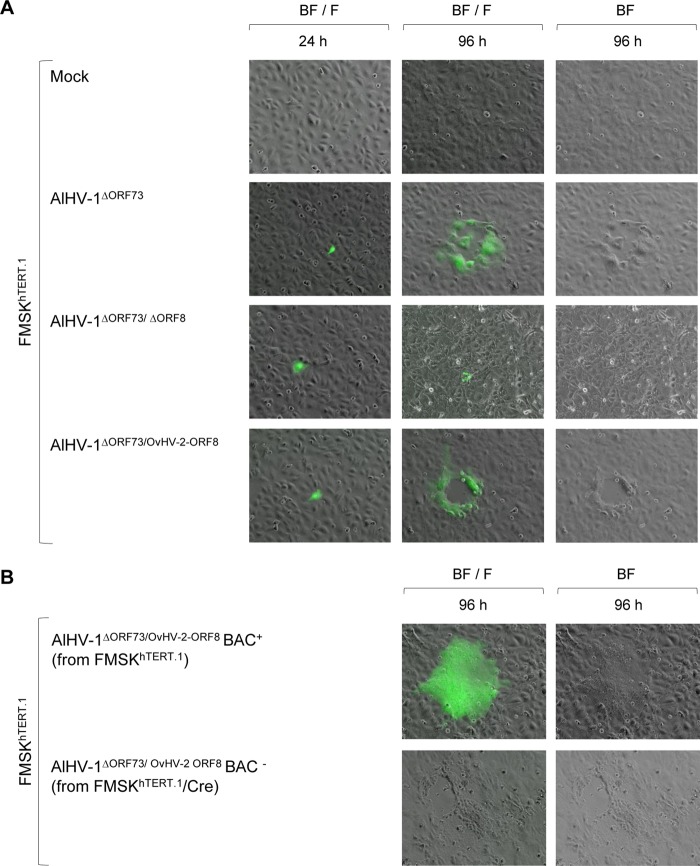
Plaque formation and viral replication in cell culture. (A) Representative fluorescence microscopy images of FMSK*^hTERT.1^* cells transfected with AlHV-1 BAC DNA from different constructs at 24 and 96 h posttransfection. Nontransfected cells (mock) were used as a control. Virus spreading and cytopathic effect are indicated by the formation of plaques. Green fluorescence indicates expression of green fluorescent protein encoded by the BAC cassette. BF, bright field; F, fluorescence with a fluorescein isothiocyanate (FITC) filter. Magnification, ×10. (B) Representative images of FMSK*^hTERT.1^* cells infected with AlHV-1^ΔORF73/OvHV-2-ORF8^ reconstituted from FMSK*^hTERT.1^* (BAC^+^, BAC cassette intact) or FMSK*^hTERT.1^*/Cre cells (BAC^−^, BAC cassette excised) at 96 h postinfection.

**FIG 3:**
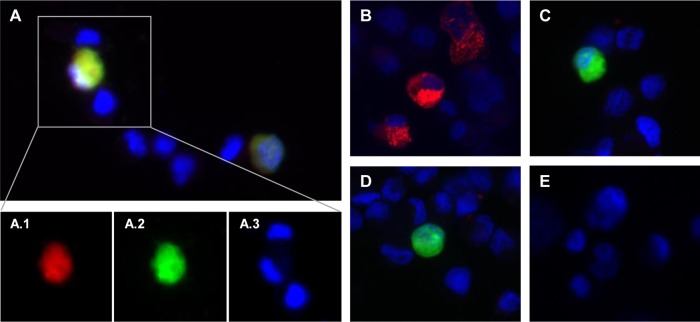
Reactivity of OvHV-2 gB-specific antibodies to the AlHV-1^ΔORF73/OvHV-2-ORF8^ virus. Representative fluorescence microscopy images of FMSK*^hTERT.1^* cells harvested at 24 h posttransfection with AlHV-1^ΔORF73/OvHV-2-ORF8^ (A and C), pOvHV-2 ORF8 (B), or AlHV-1^ΔORF73/ΔORF8^ (D) DNA and untransfected cells (E). Cells were treated with OvHV-2 gB hyperimmune mouse serum (A, B, D, and E) or preimmune serum (C) as a primary antibody and an anti-mouse IgG conjugated to Alexa Fluor 568 as a secondary antibody. Slides were mounted with SlowFade Gold Antifade Mountant with DAPI and examined using fluorescence microscopy. Individual images from the indicated area of merged image A are shown in A.1, A.2, and A.3. Red fluorescence indicates reactivity of serum antibodies with gB, green fluorescence indicates expression of green fluorescent protein encoded by the BAC cassette, and cell nuclei are stained blue. Magnification, ×20.

**FIG 4:**
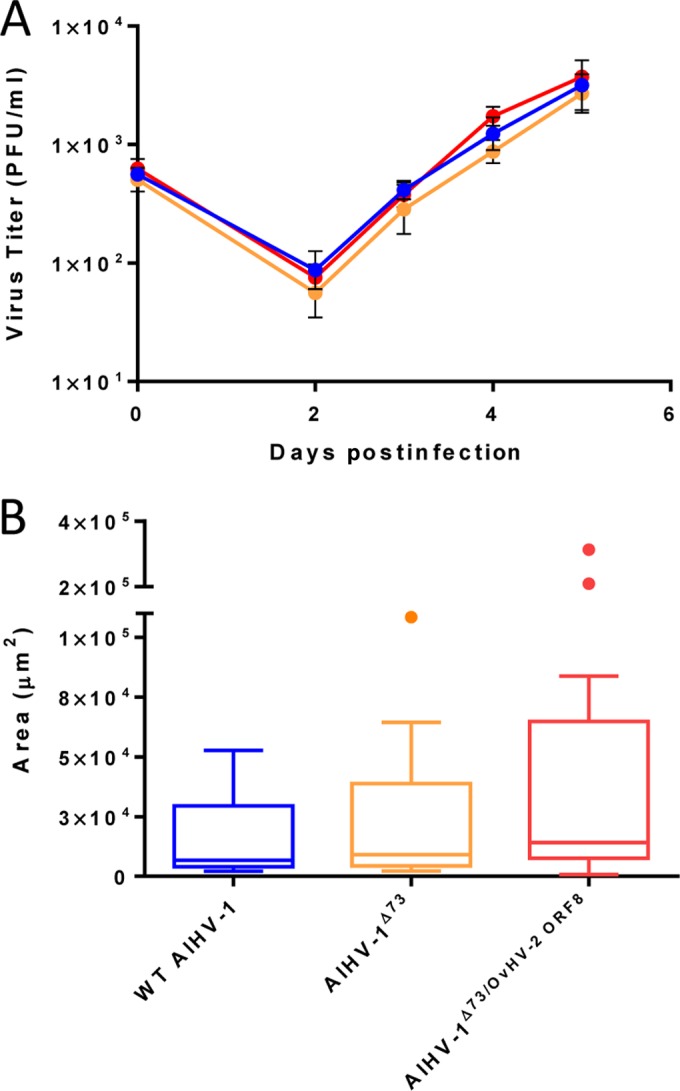
Viral replication kinetics and plaque size assay. (A) Multistep growth curves of the AlHV-1/OvHV-2 chimera (AlHV-1^ΔORF73/OvHV-2-ORF8^ [red line]), parental (AlHV-1^ΔORF73^ [orange line]), and the wild-type AlHV-1 (blue line) viruses in FMSK^hTERT.1^ cells. Virus titers at day zero were calculated from viral stock titrations. Data presented for days 2, 3, 4, and 5 are means ± standard deviations (SD) (error bars) of triplicate measurements. No statistically significant difference in titers among the three viruses was observed at any time postinoculation (*P* = 0.8270 by ANOVA). (B) Average plaque size of the chimeric, parental, and wild-type (WT) viruses measured at 3 days postinfection. Boxes indicate median plus the 25th and 75th percentiles for 14 plaques measured for each virus. Dots indicate outliers, as defined by the Tukey method. No statistically significant difference in the plaque sizes of the three viruses was observed (*P* = 0.1561 by ANOVA).

By using recombination strategies, constructs containing the AlHV-1 ORF8 gene replaced by the *galK* gene (AlHV-1^ΔORF73/ΔORF8^) or by the OvHV-2 ORF8 gene (AlHV-1^ΔORF73/OvHV-2-ORF8^) were successfully obtained, as confirmed by sequencing. Digestion of each construct and the parental bacterial artificial chromosome (BAC) DNA with two restriction enzymes, one that does not cut (SpeI) and another that cuts (EcoRI) within the recombined region (Fig. 1), yielded expected restriction patterns, confirming the correct recombination events and indicating the overall integrity of the genomes. To evaluate the ability of the AlHV-1 constructs to infect cells, BAC DNA was first transfected into immortalized fetal mouflon sheep kidney (FMSK*^hTERT.1^*) cells, and plaque formation was evaluated. As illustrated in Fig. 2A, green fluorescence resulting from the expression of green fluorescent protein (GFP) by the BAC cassette was visualized in individual cells at 24 h, indicating that BAC DNA was taken up by cells. No green fluorescence or plaques were visualized in mock transfected cells. At 96 h posttransfection, plaques in cells infected with AlHV-1^ΔORF73/OvHV-2-ORF8^ and AlHV-1^ΔORF73^ were visualized, indicating reconstitution of viral particles and cytopathic effect (CPE). Conversely, although cells were successfully transfected with DNA from the AlHV-1^ΔORF73/ΔORF8^ BAC, no plaque formation was observed at 96 h, indicating that this mutant virus was unable to spread to other cells. The nonviability of the AlHV-1 mutant that had ORF8 deleted (AlHV-1^ΔORF73/ΔORF8^) is not surprising, given that gB has been shown to be essential for virus entry in several herpesviruses (10). Even though the essentiality of gB for AlHV-1 was not investigated in this study, the finding that OvHV-2 gB can replace AlHV-1 gB in the entry process, as demonstrated in AlHV-1^ΔORF73/OvHV-2-ORF8^, is novel and useful information. Although gB from equine herpesvirus 1 (EHV-1) can be replaced by the EHV-4 homolog, gB homologs are not universally interchangeable (11–13). The loxP-flanked BAC cassette was successfully removed when AlHV-1^ΔORF73/OvHV-2-ORF8^ was cultured on FMSK*^hTERT.1^*/Cre cells, as confirmed by the absence of GFP expression (Fig. 2B). As expected, chimeric viruses with either the BAC cassette excised or intact were able to infect cells, spread, and cause CPE, as indicated by the presence of plaques at 96 h postinfection (Fig. 2B). Importantly, specific gB staining in cells infected with AlHV-1^ΔORF73/OvHV-2-ORF8^ was visualized when serum against OvHV-2 gB was used (Fig. 3A), confirming expression of OvHV-2 gB protein by the AlHV-1/OvHV-2 chimeric virus. Specificity of the antibodies used was confirmed by detection of OvHV-2 gB expressed by a plasmid (pOvHV-2 ORF8), which was used as a positive control (Fig. 3B). When treated with a negative serum, no staining was observed in cells transfected with either AlHV-1^ΔORF73/OvHV-2-ORF8^ (Fig. 3C) or pOvHV-2 ORF8 DNA (not shown). No cross-reactivity was detected in cells transfected with AlHV-1^ΔORF73/ΔORF8^ (Fig. 3D) or in untransfected cells (Fig. 3E) treated with the OvHV-2 gB-specific serum. Next, we evaluated whether replacement of the AlHV-1 ORF8 gene by the OvHV-2 homolog affects virus growth in FMSK*^hTERT.1^* cells. As illustrated in Fig. 4A, the growth kinetics of AlHV-1^ΔORF73/OvHV-2-ORF8^ were similar to those of the parental and wild-type viruses (*P* = 0.8270 by analysis of variance [ANOVA]). Plaque sizes were also similar in the three viruses (*P* = 0.1561 by ANOVA; Fig. 4B). These results indicated that the chimeric virus has the same replicative fitness as the parental virus, which is an important characteristic when considering the AlHV-1/OvHV-2 chimeric virus as a vaccine. The possibility of generating AlHV-1/OvHV-2 chimeric viruses makes available a novel way to study OvHV-2 pathogenesis by identifying proteins that may promote or restrict viral infection. Such studies are also essential to guide the development of efficacious MCF vaccines.

From a sheep-associated MCF (SA-MCF) vaccine development standpoint, the AlHV-1 expressing OvHV-2 gB is a potentially valuable tool. For insight in this direction, we used a rabbit model of infection (21) to test whether AlHV-1^ΔORF73/OvHV-2-ORF8^ is able to infect animals and induce production of antibodies without causing MCF. Rabbits were inoculated with the AlHV-1/OvHV-2 chimera, the parental AlHV-1, or the wild-type AlHV-1 by intranasal nebulization, and the outcome of infection was monitored. Infection outcomes following inoculation are summarized in Table 1. As expected, no viral DNA was detected in peripheral blood leukocytes (PBL) of rabbits inoculated with AlHV-1^ΔORF73/OvHV-2-ORF8^ or with AlHV-1^ΔORF73^; however, AlHV-1-specific antibodies were detected in serum starting at 42 days postinoculation (dpi), confirming that the animals were infected. OvHV-2 gB-specific antibodies were also detected in all four rabbits infected with the AlHV-1/OvHV-2 chimeric virus at 72 dpi; OvHV-2 gB antibody titers in these animals ranged from 40 to 80. The absence of viral DNA in the PBL after infection with the AlHV-1 ORF73-null virus has been shown previously (8). This is probably simply due to the inability of the virus to establish latent infection/episomal maintenance in lymphocytes. There is sufficient acute virus replication during the first days after infection *in vivo* to induce antibodies, and then the virus is simply cleared by the immune response and does not persist. None of the rabbits inoculated with the chimeric virus or the parental virus developed fever or any other clinical sign and were healthy at the end of the experiment. In contrast, the rabbits inoculated with wild-type AlHV-1 had detectable levels of viral DNA in PBL, starting at 35 dpi, and developed fever between 36 and 51 dpi. MCF was confirmed by the presence of AlHV-1 DNA (ranging from 1 × 10^4^ to 5 × 10^5^ viral genome copies per 50 ng of tissue total DNA) and histological lesions compatible with AlHV-1-induced MCF in all three tissues examined (lung, popliteal lymph node, and liver); perivascular infiltration of lymphoblastoid cells in the lung of one representative rabbit is illustrated in Fig. S2 in the supplemental material. In addition, three out of four rabbits in this group seroconverted to AlHV-1 between 35 and 45 dpi.

**TABLE 1:** Infection outcome following AlHV-1^ΔORF73/OvHV-2 ORF8^, AlHV-1^ΔORF73^, or wild-type AlHV-1 inoculation in rabbits^a^

Virus and rabbit	Clinical signs (dpi)^b^	AlHV-1 DNA in PBL (dpi)^c^	Antibody response		Outcome	AlHV-1 DNA in tissue (dpi)^f^		
			OvHV-2 gB (dpi)^d^	AlHV-1 (dpi)^e^		Lung	PLN	Liver
AlHV-1^ΔORF73/OvHV-2 ORF8^								
2076	−	ND	+ (72)	+ (57)	Healthy	NT	NT	NT
2077	−	ND	+ (72)	+ (72)	Healthy	NT	NT	NT
2078	−	ND	+ (72)	+ (57)	Healthy	NT	NT	NT
2079	−	ND	+ (72)	+ (42)	Healthy	NT	NT	NT
AlHV-1^ΔORF73^								
2074	−	ND	ND	+ (57)	Healthy	NT	NT	NT
2075	−	ND	ND	+ (57)	Healthy	NT	NT	NT
2076	−	ND	ND	+ (42)	Healthy	NT	NT	NT
2079	−	ND	ND	+ (57)	Healthy	NT	NT	NT
Wild-type AlHV-1								
2083	+ (36)	+ (35)	ND	+ (42)	MCF	1 × 10^4^ (36)	3 × 10^4^ (36)	1 × 10^4^ (36)
2084	+ (50)	+ (45)	ND	+ (42)	MCF	4 × 10^4^ (52)	3 × 10^5^ (52)	1 × 10^4^ (52)
2085	+ (50)	+ (42)	ND	+ (42)	MCF	1 × 10^5^ (50)	5 × 10^5^ (50)	9 × 10^4^ (50)
2086	+ (51)	+ (50)	ND	ND	MCF	4 × 10^4^ (52)	3 × 10^5^ (52)	1 × 10^5^ (52)

a The values in parentheses in the table show the time (days postinoculation [dpi]) when the clinical sign, DNA, or antibody response was observed.

b Clinical signs shown as follows: −, no clinical signs; +, presence of clinical signs (fever).

c AlHV-1 DNA detected in peripheral blood leukocytes (PBL). ND, AlHV-1 DNA not detected at any time point throughout the experiment; +, AlHV-1 DNA detected by PCR.

d +, anti-OvHV-2 gB antibodies detected by ELISA; ND, not detected.

e +, anti-AlHV-1 antibodies detected by ELISA; ND, not detected.

f AlHV-1 genome copy number per 50 ng of total DNA in tissue as quantified by qPCR; PLN, popliteal lymph nodes; NT, not tested.

Together, these results show that AlHV-1^ΔORF73/OvHV-2-ORF8^ was nonpathogenic for rabbits at the dose used (2.1 × 10^4^ PFU) but sufficient to stimulate the production of antibodies specific to OvHV-2 gB; in contrast, the wild-type AlHV-1 caused MCF in all challenged rabbits. Infection parameters, such as viral DNA in PBL and disease development, observed in this study were consistent with previous data reported for the wild-type and AlHV-1^ΔORF73^ viruses (8). It is important to note that even using a lower inoculum of AlHV-1^ΔORF73/OvHV-2-ORF8^ (2.1 × 10^4^ PFU) than the wild-type virus inoculum (3.2 × 10^4^ PFU), the chimeric virus dose was enough to induce antibodies against the recombinant protein, which is an essential characteristic regarding the use of this construction as a vaccine. Although the experiment described here was not appropriate for challenge studies due to the small number of animals used, it demonstrated that anti-gB antibody responses were stimulated in the animals infected with AlHV-1^ΔORF73/OvHV-2-ORF8^ and paved the way for future experiments aimed at evaluating this chimeric virus as a vaccine against SA-MCF. The nonpathogenic AlHV-1 used as a backbone in this study is an attractive alternative to express OvHV-2 gB in the lung, the site of initial infection where production of neutralizing antibodies is needed.

Another drawback caused by the inability to propagate OvHV-2 *in vitro* is that there is no neutralization assay available for this virus. We have previously developed an *in vivo* system to measure neutralizing activity of antibodies against OvHV-2 (14). Although this assay allowed the identification of neutralizing antibodies capable of blocking OvHV-2 infection and the evaluation of cross-reactivity of neutralizing antibodies between AlHV-1 and OvHV-2 (7), it is not practical to assess neutralizing antibody responses on a large scale following vaccination. For that, an *in vitro* OvHV-2 neutralization assay is needed. In this study, we evaluated the AlHV-1/OvHV-2 chimeric virus in a neutralization assay for OvHV-2 gB. AlHV-1^ΔORF73/OvHV-2-ORF8^ infection of FMSK*^hTERT.1^* cells was blocked by OvHV-2-specific antibodies with 50% tissue culture infective dose (TCID_50_) titers that ranged from 8 to 256, while no neutralization activity was observed when negative sera were used (Table 2). These results indicate that a neutralization assay based on this chimeric virus can be used for detection and quantification of neutralizing antibodies specific for the heterologous protein and prompt future studies to validate the assay.

**TABLE 2:** Blocking of AlHV-1^ΔORF73/OvHV-2-ORF8^ by OvHV-2-specific antibodies from rabbits and sheep

OvHV-2 serology^a^	Sample^b^	Neutralization titer (TCID_50_)
Positive	Rabbit 1 (IM)	128
	Rabbit 2 (IM)	256
	Sheep 1 (ExI)	32
	Sheep 2 (ExI)	128
	Sheep 3 (NaI)	8
	Sheep 4 (NaI)	64
Negative	Rabbit 1 (pre)	<8
	Rabbit 2 (pre)	<8
	Rabbit 3 (NI)	<8
	Sheep 5 (NI)	<8
	Sheep 6 (NI)	<8
	Sheep 7 (NI)	<8

a OvHV-2 specific antibodies as tested by MCFV cELISA.

b The time and treatment of the animal is shown in parentheses as follows: pre, pre immunization; IM, OvHV-2 gB immunized; ExI, experimental OvHV-2 infection; NaI, natural OvHV-2 infection; NI, noninfected/immunized.

In summary, this study shows that the replacement of the AlHV-1 gB-encoding gene by its OvHV-2 homolog did not affect the ability of AlHV-1 to infect and spread in mammalian cells and that the constructed virus, AlHV-1^ΔORF73/OvHV-2-ORF8^, was nonpathogenic for rabbits and induced antibody response against the OvHV-2 gB protein. Together, these data suggest that the AlHV-1/OvHV-2 chimeric virus is an attractive alternative to be tested as a vaccine for SA-MCF. In addition, because anti-OvHV-2 antibodies can neutralize AlHV-1^ΔORF73/OvHV-2-ORF8^, this chimeric virus is a valuable tool for the detection and quantification of OvHV-2 gB neutralizing antibodies *in vitro*, which is also critical for vaccine development.

## 

### AlHV-1/OvHV-2 chimeric virus.

AlHV-1^ΔORF73^ BAC (8) was used as the backbone for construction of the chimeric virus. The *galK* recombineering system (15), performed according to protocols available at http://redrecombineering.ncifcrf.gov, was used to manipulate the viral genome in the BAC. A schematic representation of the recombineering steps and the oligonucleotides used to replace the AlHV-1 ORF8, encoding gB, by the OvHV-2 homologous gene are presented in Fig. 1 and Table 3, respectively. Specifically, a *galK* gene sequence flanked by arms R1 and R2, corresponding to the AlHV-1 gene to be replaced (AlHV-1 ORF8), was produced by PCR using the primers R1-*galK*-F and R2-*galK*-R and a plasmid containing the *galK* gene as the template. The fragment obtained, R1-*galK*-R2, was transformed into *Escherichia coli* SW102 containing the AlHV-1^ΔORF73^ BAC. A clone containing an ORF8-deleted AlHV-1 BAC (AlHV-1^ΔORF73/ΔORF8^) was positively selected using galactose. Next, an AlHV-1^ΔORF73/ΔORF8^ clone was subjected to recombination with a DNA fragment containing the OvHV-2 ORF8 gene flanked by the R1 and R2 arms. This fragment was produced by PCR using primers R1-OvHV-2-ORF8-F and R2-OvHV-2-ORF8-R and a plasmid containing a codon-optimized sequence of the OvHV-2 ORF8 gene (see Fig. S1 in the supplemental material) as the template. Negative selection was performed on plates containing minimal medium and 0.4% 2-deoxygalactose. Clones containing the OvHV-2 ORF8 gene were screened by PCR using primers Pre-R1-F and Post-R2-R. BAC DNA was purified using NucleoBond BAC100 (Clontech) per the manufacturer’s recommendations. The authenticity and integrity of recombinant clones were verified by both sequencing and digestion pattern using SpeI or EcoRI (New England BioLabs Inc.).

**TABLE 3:** Oligonucleotides used in the study

Oligonucleotide ID^a^	Sequence (5′ to 3′)^b^
R1 region	TAGTTTATAGCAACAAAAAGTGGATATTTAAAGACTTGTATGCCCTCCTGTACGCCCACATGCAACTAGCCAACA
R2 region	AATTTACAAACATAGTGAGTCATACACCAGGAAGTCAGTACAACATGCTCTTACAATGAGTCATACACTTTATTA
R1-*galK*-F	TAGTTTATAGCAACAAAAAGTGGATATTTAAAGACTTGTATGCCCTCCTGTACGCCCACATGCAACTAGCCAACACCTGTTGACAATTAATCATCGGCA
R2-*galK*-R	AATTTACAAACATAGTGAGTCATACACCAGGAAGTCAGTACAACATGCTCTTACAATGAGTCATACACTTTATTATCAGCACTGTCCTGCTCCTT
R1-OvHV-2-ORF8-F	TAGTTTATAGCAACAAAAAGTGGATATTTAAAGACTTGTATGCCCTCCTGTACGCCCACATGCAACTAGCCAACAATGGCTTCTCCTACCTCTACC
R2-OvHV-2-ORF8-R	AATTTACAAACATAGTGAGTCATACACCAGGAAGTCAGTACAACATGCTCTTACAATGAGTCATACACTTTATTACAGGGCAGCAGCAGACTC
Pre-R1-F	CCACTGCTTGCTCATCG
Post-R2-R	AAAGGCAAGGTTGTAATG

a ID, identification; F, forward; R, reverse.

b Arms R1 and R2 sequences are underlined.

10.1128/mSphere.00108-16.1 Figure S1  OvHV-2 ORF8 sequence codon optimized for mammalian expression. Download Figure S1, PDF file, 0.1 MB. Copyright © 2016 Cunha et al. 2016 Cunha et al. This content is distributed under the terms of the Creative Commons Attribution 4.0 International license.

10.1128/mSphere.00108-16.2 Figure S2  Typical AlHV-1-induced lymphoproliferation in lung, associated with a vein exhibiting endophlebitis (arrow). This histopathological lesion is representative of all rabbits infected with the wild-type AlHV-1. Image A was taken at a magnification of ×100. Image B is a magnification of the squared area of image A. Download Figure S2, PDF file, 0.2 MB. Copyright © 2016 Cunha et al. 2016 Cunha et al. This content is distributed under the terms of the Creative Commons Attribution 4.0 International license.

### Cells.

FMSK cells were maintained in complete Dulbecco’s modified Eagle medium (complete DMEM) (DMEM supplemented with 10% fetal bovine serum, 100 U/ml penicillin, 100 µg/ml streptomycin, and 1 µg/ml amphotericin B) at 37°C in 5% CO_2_.

To generate an immortalized cell line, FMSK cells were stably transfected with the plasmid pBABE-puro-*hTERT* (16) using Attractene transfection reagent (Qiagen). Cells were selected using puromycin (3 µg/ml) for 12 days and then cloned by limiting dilution with continued selection in puromycin (2 µg/ml). The cell line was designated FMSK*^hTERT.1^*.

To generate cells expressing Cre recombinase, FMSK*^hTERT.1^* cells were transfected with the plasmid pEFIN3-NLS-Cre (17) using Lipofectamine LTX with Plus reagent (ThermoFisher Scientific). Stably transfected cells, designated FMSK^hTERT.1^/Cre, were selected using Geneticin (150 µg/ml) and used without further cloning.

### Virus reconstitution.

FMSK*^hTERT.1^* cells (1 × 10^5^/well of 12-well plates) were transfected with BAC DNA (1.25 µg) using Lipofectamine LTX with Plus reagent (ThermoFisher Scientific). Fluorescence microscopy was used to assess successful transfections by visualization of GFP. Five days posttransfection, virus was expanded by passaging infected cells with uninfected FMSK*^hTERT.1^* cells. Infected cells were frozen at −80°C when the CPE was about 80%. Virus stocks were prepared by freezing and thawing infected cells three times. Supernatant containing virus was stored at −80°C until use.

The titer of virus stocks was calculated using a standard plaque assay. Briefly, virus was serially diluted in DMEM and incubated with FMSK*^hTERT.1^* for 2 h at 37°C. Cells were washed and overlaid with 2% carboxymethyl cellulose in DMEM with 1% FBS. The number of PFU was counted at 5 dpi after fixation (10% formaldehyde) and staining (0.1% crystal violet).

To excise the loxP-flanked BAC cassette and expand virus stocks, FMSK^hTERT.1^/Cre cells were infected with virus at a multiplicity of infection (MOI) of approximately 0.6. Infected cells were mixed with uninfected FMSK^hTERT.1^/Cre cells at 10 dpi, and virus stocks were prepared as described above. Loss of GFP expression in viral plaques was monitored by fluorescence microscopy.

### Growth curves and plaque size assay.

Triplicate cultures of FMSK*^hTERT.1^* cells were infected with wild-type AlHV-1, AlHV-1^ΔORF73^, or AlHV-1^ΔORF73/OvHV-2-ORF8^ at an MOI of 0.001. Virus was incubated with cells for 2 h at 37°C and then replaced by complete DMEM. Cells and supernatants were collected at various time points postinfection and stored at −80°C. Infectious virus was quantitated in triplicate as described above. The mean number of PFU of each virus per milliliter was compared using ANOVA; a *P* value of ≤0.05 was considered statistically significant.

For plaque size measurement, live cells were examined for GFP expression at 3 days postinfection using a Nikon Eclipse Ti-S microscope equipped with a Digital Sight Qi7Mc camera. The perimeters of viral plaques were outlined freehand, and the pixels were converted to actual dimensions using the NIS-Elements Basic Research software (ver. 3.06, Nikon). The mean area of plaques of each virus was compared using ANOVA; a *P* value of ≤0.05 was considered statistically significant.

### Indirect immunofluorescence staining.

FMSK*^hTERT.1^* cells were transfected with AlHV-1^ΔORF73/OvHV-2-ORF8^, AlHV-1^ΔORF73/ΔORF8^, or a plasmid expressing OvHV-2 gB (pOvHV-2 ORF8) and cultured as described above. Following 24 h in culture, transfected and untransfected cells were washed in phosphate-buffered saline (PBS) (pH 7.4) and lifted using 10 mM EDTA (5 min at 37°C). Cells were immobilized on glass slides and fixed/permeabilized in 25% methanol−75% acetone. For immunostaining, a homemade mouse hyperimmune serum against OvHV-2 gB (1:80) or the mouse preimmune serum (1:80) were used. For detection, a goat anti-mouse IgG (H+L) Alexa Fluor 568 conjugate (ThermoFisher Scientific) was used (1:1,000). Slides were mounted with SlowFade Gold Antifade Mountant with DAPI (4′,6′-diamidino-2-phenylindole) (ThermoFisher Scientific), and cells were visualized by epifluorescence microscopy.

### Rabbit infection.

Three-month-old New Zealand rabbits were used in this study. The animals were maintained at Washington State University, Pullman, WA, in accordance with approved animal care and use protocols. Four rabbits were inoculated by intranasal nebulization with the AlHV-1/OvHV-2 chimeric virus (AlHV-1^ΔORF73/OvHV-2-ORF8^, 2.1 × 10^4^ PFU/rabbit), four rabbits were nebulized with the AlHV-1 parental virus (AlHV-1^ΔORF73^, 3.2 × 10^4^ PFU/rabbit), and four rabbits were nebulized with the wild-type AlHV-1 (C500 strain, 3.2 × 10^4^ PFU/rabbit). The AlHV-1^ΔORF73/OvHV-2-ORF8^ and AlHV-1^ΔORF73^ viruses used in this experiment had the BAC cassette excised. Animals were regularly monitored for clinical signs of MCF and tested for AlHV-1 DNA and specific antibodies in blood. AlHV-1 DNA was detected and quantified by quantitative PCR (qPCR) (18). Antibodies specific for OvHV-2 gB or MCF viruses antibodies were detected by enzyme-linked immunosorbent assay (ELISA) or competitive ELISA (cELISA), respectively, as previously described (9, 19). Animals that developed clinical signs were euthanized within 48 h of onset of fever (≥40°C) and necropsied immediately. The lungs, liver, and popliteal lymph nodes were collected and processed for confirmation of MCF based on histopathology and detection of AlHV-1 DNA. Euthanasia and postmortem examination and testing were performed as previously described (7, 20). Healthy animals were maintained for 73 dpi, when the experiment was terminated.

### Neutralization assay.

AlHV-1^ΔORF73/OvHV-2-ORF8^ (10^2^ TCID_50_) was incubated with serially diluted sera for 1 h at 37°C and then mixed with FMSK*^hTERT.1^* cells. The virus, serum, and cells (2.5 × 10^4^ cells/well) mixture was seeded into 96-well plates and incubated for 5 days. The titer of neutralizing antibodies against OvHV-2 gB was the reciprocal of the highest serum dilution that inhibited CPE in ≥50% of the wells. Tested samples included homemade hyperimmune sera from rabbits (rabbits 1 and 2, immunized with OvHV-2 gB DNA [9]) and sera from sheep experimentally or naturally infected with OvHV-2. Serum samples from naive animals (preimmune or uninfected) were used as negative controls. All sera were confirmed positive or negative to anti-MCF viruses gB antibodies by cELISA (19).

## References

[1] Davison AJ, Eberle R, Ehlers B, Hayward GS, McGeoch DJ, Minson AC, Pellett PE, Roizman B, Studdert MJ, Thiry E 2009 The order Herpesvirales. Arch Virol 154:171–177. doi:10.1007/s00705-008-0278-4. 19066710 PMC3552636

[2] O’Toole D, Li H 2014 The pathology of malignant catarrhal fever, with an emphasis on ovine herpesvirus 2. Vet Pathol 51:437–452. doi:10.1177/0300985813520435. 24503439

[3] Li H, Cunha CW, Taus NS, Knowles DP 2014 Malignant catarrhal fever: inching toward understanding. Annu Rev Anim Biosci 2:209–233. doi:10.1146/annurev-animal-022513-114156. 25384141

[4] Dry I, Haig DM, Inglis NF, Imrie L, Stewart JP, Russell GC 2008 Proteomic analysis of pathogenic and attenuated alcelaphine herpesvirus 1. J Virol 82:5390–5397. doi:10.1128/JVI.00094-08. 18353942 PMC2395210

[5] Haig DM, Grant D, Deane D, Campbell I, Thomson J, Jepson C, Buxton D, Russell GC 2008 An immunisation strategy for the protection of cattle against alcelaphine herpesvirus-1-induced malignant catarrhal fever. Vaccine 26:4461–4468. doi:10.1016/j.vaccine.2008.06.056. 18601965

[6] Russell GC, Benavides J, Grant D, Todd H, Deane D, Percival A, Thomson J, Connelly M, Haig DM 2012 Duration of protective immunity and antibody responses in cattle immunised against alcelaphine herpesvirus-1-induced malignant catarrhal fever. Vet Res 43:51. doi:10.1186/1297-9716-43-51. 22686373 PMC3425131

[7] Taus NS, Cunha CW, Marquard J, O’Toole D, Li H 2015 Cross-reactivity of neutralizing antibodies among malignant catarrhal fever viruses. PLoS One 10:e00108-16. doi:10.1371/journal.pone.0145073. PMC4681746 26658281

[8] Palmeira L, Sorel O, Van Campe W, Boudry C, Roels S, Myster F, Reschner A, Coulie PG, Kerkhofs P, Vanderplasschen A, Dewals BG 2013 An essential role for γ-herpesvirus latency-associated nuclear antigen homolog in an acute lymphoproliferative disease of cattle. Proc Natl Acad Sci U S A 110:E1933–E1942. doi:10.1073/pnas.1216531110. 23630278 PMC3666693

[9] Cunha CW, Knowles DP, Taus NS, O’Toole D, Nicola AV, Aguilar HC, Li H 2015 Antibodies to ovine herpesvirus 2 glycoproteins decrease virus infectivity and prevent malignant catarrhal fever in rabbits. Vet Microbiol 175:349–355. doi:10.1016/j.vetmic.2014.11.026. 25542288

[10] Eisenberg RJ, Atanasiu D, Cairns TM, Gallagher JR, Krummenacher C, Cohen GH 2012 Herpesvirus fusion and entry: a story with many characters. Viruses 4:800–832. doi:10.3390/v4050800. 22754650 PMC3386629

[11] Lee SK, Compton T, Longnecker R 1997 Failure to complement infectivity of EBV and HSV-1 glycoprotein B (gB) deletion mutants with gBs from different human herpesvirus subfamilies. Virology 237:170–181. doi:10.1006/viro.1997.8765. 9344919

[12] Miethke A, Keil GM, Weiland F, Mettenleiter TC 1995 Unidirectional complementation between glycoprotein B homologues of pseudorabies virus and bovine herpesvirus 1 is determined by the carboxy-terminal part of the molecule. J Gen Virol 76:1623–1635. doi:10.1099/0022-1317-76-7-1623. 9049369

[13] Spiesschaert B, Osterrieder N, Azab W 2015 Comparative analysis of glycoprotein B (gB) of equine herpesvirus type 1 and type 4 (EHV-1 and EHV-4) in cellular tropism and cell-to-cell transmission. Viruses 7:522–542. doi:10.3390/v7020522. 25654240 PMC4353902

[14] Li H, Cunha CW, O’Toole D, Nicola AV, Knowles DP, Taus NS 2013 Development of an *in vivo* system to measure antibody-blocking of ovine herpesvirus 2 entry. J Virol Methods 188:104–107. doi:10.1016/j.jviromet.2012.12.008. 23274755

[15] Warming S, Costantino N, Court DL, Jenkins NA, Copeland NG 2005 Simple and highly efficient BAC recombineering using galK selection. Nucleic Acids Res 33:e36. 15731329 10.1093/nar/gni035 PMC549575

[16] Counter CM, Hahn WC, Wei W, Caddle SD, Beijersbergen RL, Lansdorp PM, Sedivy JM, Weinberg RA 1998 Dissociation among *in vitro* telomerase activity, telomere maintenance, and cellular immortalization. Proc Natl Acad Sci U S A 95:14723–14728. doi:10.1073/pnas.95.25.14723. 9843956 PMC24516

[17] Gillet L, Daix V, Donofrio G, Wagner M, Koszinowski UH, China B, Ackermann M, Markine-Goriaynoff N, Vanderplasschen A 2005 Development of bovine herpesvirus 4 as an expression vector using bacterial artificial chromosome cloning. J Gen Virol 86:907–917. doi:10.1099/vir.0.80718-0. 15784885

[18] Traul DL, Elias S, Taus NS, Herrmann LM, Oaks JL, Li H 2005 A real-time PCR assay for measuring alcelaphine herpesvirus-1 DNA J Virol Methods 129:186–190. doi:10.1016/j.jviromet.2005.05.021. 15998545

[19] Li H, McGuire TC, Müller-Doblies UU, Crawford TB 2001 A simpler, more sensitive competitive inhibition ELISA for detection of antibody to malignant catarrhal fever viruses. J Vet Diagn Invest 13:361–364. 11478614 10.1177/104063870101300417

[20] Dewals B, Myster F, Palmeira L, Gillet L, Ackermann M, Vanderplasschen A 2011 Ex vivo bioluminescence detection of alcelaphine herpesvirus 1 infection during malignant catarrhal fever. J Virol 85:6941–6954. doi:10.1128/JVI.00286-11. 21593175 PMC3126570

[21] Cunha CW, O'Toole D, Taus NS, Knowles DP, Li H 2013 Are rabbits a suitable model to study sheep-associated malignant catarrhal fever in susceptible hosts? Vet Microbiol 163:358–363. doi:10.1016/j.vetmic.2013.01.002. 23394795

