# Supplementary material for: Replacement of Glycoprotein B in Alcelaphine Herpesvirus 1 by Its Ovine Herpesvirus 2 Homolog : Implications in Vaccine Development for Sheep-Associated Malignant Catarrhal Fever
Source: mSphere. 2016 Aug 3;1(4):e00108-16. doi: 10.1128/mSphere.00108-16 (PMC4973634; doi:10.1128/mSphere.00108-16)
Supplement: Figure S1 [file sph004162124sf1.pdf]

Codon optimized OvHV-2 ORF8

```
          M  A  S  P   T  S  T   L  S  P   A  A  L  T   A  L  L   C  L  A   A  C  L  G   Q  T  P   T  P  P   T  T  E
   1     ATGGCTTCTC CTACCTCTAC CCTGTCTCCT GCTGCTCTGA CCGCTCTGCT GTGTCTGGCT GCTTGTCTGG GCCAGACCCC TACCCCTCCT ACCACCGAGG
         TACCGAAGAG GATGGAGATG GGACAGAGGA CGACGAGACT GGCGAGACGA CACAGACCGA CGAACAGACC CGGTCTGGGG ATGGGGAGGA TGGTGGCTCC

         D  D  V  I   V  P  G   H  T  V  S   P  D  V   L  K  Q   Q  L  G  S   G  L  N   E  E  E   G  S  I  N   R  G  P
 101     ATGATGTGAT CGTGCCTGGC CACACCGTGT CTCCAGATGT GCTGAAGCAG CAGCTGGGCT CTGGCCTGAA TGAGGAGGAG GGCTCTATCA ATAGAGGCCC
         TACTACACTA GCACGGACCG GTGTGGCACA GAGGTCTACA CGACTTCGTC GTCGACCCGA GACCGGACTT ACTCCTCCTC CCGAGATAGT TATCTCCGGG

           M  D  P   S  A  F  P   F  R  V   C  S  A   S  N  I  G   D  I  F   R  F  Q   T  T  H  S   C  P  N   T  K  D
 201     TATGGATCCT TCTGCCTTCC CTTTCAGAGT GTGCTCTGCT TCTAACATCG GCGACATCTT CAGGTTCCAG ACCACCCACT CTTGTCCTAA CACCAAGGAT
         ATACCTAGGA AGACGGAAGG GAAAGTCTCA CACGAGACGA AGATTGTAGC CGCTGTAGAA GTCCAAGGTC TGGTGGGTGA GAACAGGATT GTGGTTCCTA

          K  E  H  N   E  G  I   L  L  I   F  K  E  N   I  V  P   Y  V  F   K  V  R  K   Y  R  K   I  V  T   T  S  T
 301     AAGGAGCACA ACGAGGGCAT CCTGCTGATC TTCAAGGAGA ACATCGTCCC CTACGTGTTC AAGGTGCGGA AGTACAGAAA GATCGTGACC ACCTCTACCG
         TTCCTCGTGT TGCTCCCGTA GGACGACTAG AAGTTCCTCT TGTAGCAGGG GATGCACAAG TTCCACGCCT TCATGTCTTT CTAGCACTGG TGGAGATGGC

         V  Y  N  G   I  Y  S   D  A  I  T   N  Q  H   E  F  S   K  S  V  P   H  Y  E   A  R  R   M  D  T  I   Y  Q  C
 401     TGTACAACGG CATCTACTCT GATGCCATCA CCAACCAGCA CGAGTTCTCT AAGTCTGTGC CTCACTACGA GGCCAGAAGA ATGGATACCA TCTACCAGTG
         ACATGTTGCC GTAGATGAGA CTACGGTAGT GGTTGGTCGT GCTCAAGAGA TTCAGACACG GAGTGATGCT CCGGTCTTCT TACCTATGGT AGATGGTCAC

           Y  N  S   L  S  L  T   V  G  G   N  L  L   A  Y  T  D   N  D  G   Y  N  L   T  V  D  L   Q  P  M   D  G  L
 501     CTACAACTCC CTGTCTCTGA CCGTGGGCGG CAATCTGCTG GCTTATACCG ATAACGATGG CTACAACCTG ACCGTGGATC TGCAGCCTAT GGATGGCCTG
         GATGTTGAGG GACAGAGACT GGCACCCGCC GTTAGACGAC CGAATATGGC TATTGCTACC GATGTTGGAC TGGCACCTAG ACGTCGGATA CCTACCGGAC

          S  N  S  V   R  R  Y   N  S  Q   P  E  I  H   A  E  P   G  W  L   L  G  G  Y   R  R  R   T  T  V   N  C  E
 601     TCTAACTCCG TGAGAAGATA CAACTCTCAG CCTGAGATCC ACGCTGAGCC TGGCTGGCTG CTGGGCGGCT ACCGGAGAAG AACCACCGTG AATTGTGAGG
         AGATTGAGGC ACTCTTCTAT GTTGAGAGTC GGACTCTAGG TGCGACTCGG ACCGACCGAC GACCCGCCGA TGGCCTCTTC TTGGTGGCAC TTAACACTCC

         V  T  D  T   E  A  R   S  V  P  P   F  R  Y   F  V  T   N  V  G  D   T  I  E   M  S  P   F  W  S  G   G  A  N
 701     TGACCGATAC CGAGGCTAGA TCTGTGCCTC CTTTCAGATA CTTCGTGACC AATGTGGGCG ACACAATCGA GATGTCTCCA TTTTGGTCTG GCGGCGCTAA
         ACTGGCTATG GCTCCGATCT AGACACGGAG GAAAGTCTAT GAAGCACTGG TTACACCCGC TGTGTTAGCT CTACAGAGGT AAAACCAGAC CGCCGCGATT

           E  T  E   P  N  K  E   P  R  R   T  V  S   V  L  R  D   Y  T  L   V  D  Y   K  D  R  G   S  R  P   Q  P  H
 801     TGAGACAGAG CCTAATAAGG AGCCTCGGAG AACAGTCTCT GTGCTGAGAG ATTACACCCT GGTGGACTAC AAGGACAGAG GCTCTAGACC TCAGCCTCAC
         ACTCTGTCTC GGATTATTCC TCGGAGCCTC TTGTCAGAGA CACGACTCTC TAATGTGGGA CCACCTGATG TTCCTGTCTC CGAGATCTGG AGTCGGAGTG

          T  R  I  F   I  D  K   E  D  Y   T  L  S  W   A  Q  Q   L  K  N   I  S  Y  C   R  W  A   H  W  K   S  F  H
 901     ACCAGAATCT TCATCGACAA GGAGGACTAC ACCCTGTCTT GGGCTCAGCA GCTGAAGAAC ATCTCTTACT GTAGATGGGC CCACTGGAAG TCTTTCCACA
         TGGTCTTAGA AGTAGCTGTT CCTCCTGATG TGGGACAGAA CCCGAGTCGT CGACTTCTTG TAGAGAATGA CATCTACCCG GGTGACCTTC AGAAAGGTGT

         N  A  I  K   T  E  H   E  N  S  Y   H  F  V   A  N  D   I  T  A  S   F  F  T   P  N  T   E  A  Q  D   V  T  K
1001     ATGCTATCAA GACCGAGCAC GAGAACTCCT ACCACTTCGT GGCTAATGAT ATCACCGCCA GCTTTTTCAC CCCTAATACC GAGGCTCAGG ATGTGACCAA
         TACGATAGTT CTGGCTCGTG CTCTTGAGGA TGGTGAAGCA CCGATTACTA TAGTGGCGGT CGAAAAAGTG GGGATTATGG CTCCGAGTCC TACACTGGTT

           T  H  T   C  L  N  S   L  I  E   S  E  M   T  S  R  L   E  K  V   N  G  T   H  V  T  N   G  S  R   Q  Y  Y
1101     GACCCACACC TGTCTGAATT CCCTGATCGA GTCTGAGATG ACCTCCAGAC TGGAGAAGGT GAATGGCACC CACGTGACCA ATGGCTCTAG ACAGTACTAC
         CTGGGTGTGG ACAGACTTAA GGGACTAGCT CAGACTCTAC TGGAGGTCTG ACCTCTTCCA CTTACCGTGG GTGCACTGGT TACCGAGATC TGTCATGATG

          L  T  N  G   G  L  L   L  V  W   Q  P  L  V   Q  Q  K   L  L  N   A  Q  D  L   L  E  A   V  A  S   K  H  N
1201     CTGACCAATG GCGGCCTGCT GCTGGTGTGG CAGCCTCTGG TGCAGCAGAA GCTGCTGAAC GCTCAGGATC TGCTGGAGGC TGTGGCTTCT AAGCACAATG
         GACTGGTTAC CGCCGGACGA CGACCACACC GTCGGAGACC ACGTCGTCTT CGACGACTTG CGAGTCCTAG ACGACCTCCG ACACCGAAGA TTCGTGTTAC

         V  T  K  P   A  R  G   R  R  Q  R   R  A  V   S  S  I   L  I  D  D   D  V  Y   T  A  E   S  A  L  L   L  T  Q
1301     TGACCAAGCC TGCTAGAGGC CGGAGACAGA GAAGGGCTGT GTCCTCTATC CTGATCGACG ATGATGTGTA CACCGCTGAG TCTGCTCTGC TGCTGACCCA
         ACTGGTTCGG ACGATCTCCG GCCTCTGTCT CTTCCCGACA CAGGAGATAG GACTAGCTGC TACTACACAT GTGGCGACTC AGACGAGACG ACGACTGGGT

           I  Q  F   A  Y  D  M   L  R  S   Q  I  N   T  V  L  E   E  L  S   R  A  W   C  R  E  Q   H  R  A   S  L  M
1401     GATCCAGTTT GCTTACGACA TGCTGAGGTC CCAGATCAAT ACCGTGCTGG AGGAGCTGTC TAGAGCTTGG TGTAGAGAGC AGCATAGAGC TTCCCTGATG
         CTAGGTCAAA CGAATGCTGT ACGACTCCAG GGTCTAGTTA TGGCACGACC TCCTCGACAG ATCTCGAACC ACATCTCTCG TCGTATCTCG AAGGGACTAC

          W  N  E  L   S  K  I   N  P  T   S  V  M  S   S  I  Y   G  R  P   V  S  A  K   R  I  G   D  V  I   S  V  S
1501     TGGAACGAGC TGTCCAAGAT CAATCCTACC TCCGTGATGT CCTCCATCTA CGGCAGACCT GTGTCTGCTA AGAGAATCGG CGACGTGATC TCTGTGTCTC
         ACCTTGCTCG ACAGGTTCTA GTTAGGATGG AGGCACTACA GGAGGTAGAT GCCGTCTGGA CACAGACGAT TCTCTTAGCC GCTGCACTAG AGACACAGAG

         H  C  V  V   V  D  Q   Q  S  V  S   L  H  R   N  M  R   V  P  G  R   D  H  A   H  E  C   Y  S  R  P   P  V  T
1601     ACTGTGTGGT GGTGGATCAG CAGTCTGTGT CTCTGCACAG AAACATGAGA GTGCCTGGCA GAGATCACGC TCACGAGTGT TACTCTAGAC CTCCTGTGAC
         TGACACACCA CCACCTAGTC GTCAGACACA GAGACGTGTC TTTGTACTCT CACGGACCGT CTCTAGTGCG AGTGCTCACA ATGAGATCTG GAGGACACTG

           F  K  F   I  N  D  S   H  L  Y   K  G  Q   L  G  V  N   N  E  I   L  L  T   T  T  A  L   E  V  C   H  E  N
1701     CTTCAAGTTC ATCAACGACT CCCACCTGTA CAAGGGCCAG CTGGGCGTGA ATAATGAGAT CCTGCTGACC ACCACCGCTC TGGAGGTGTG TCACGAGAAT
         GAAGTTCAAG TAGTTGCTGA GGGTGGACAT GTTCCCGGTC GACCCGCACT TATTACTCTA GGACGACTGG TGGTGGCGAG ACCTCCACAC AGTGCTCTTA

          T  E  H  Y   F  Q  G   G  N  N   M  Y  F  Y   K  N  Y   R  H  V   K  T  I  P   V  S  A   V  A  T   L  D  T
1801     ACCGAGCATT ACTTTCAGGG CGGCAACAAC ATGTACTTCT ACAAGAACTA CCGGCACGTG AAAACCATCC CTGTGTCTGC TGTGGCTACA CTGGATACCT
         TGGCTCGTAA TGAAAGTCCC GCCGTTGTTG TACATGAAGA TGTTCTTGAT GGCCGTGCAC TTTTGGTAGG GACACAGACG ACACCGATGT GACCTATGGA

         F  I  V  L   N  L  T   L  V  E  N   I  D  F   Q  V  I   E  L  Y  S   R  E  E   K  R  M   S  T  V  F   D  I  E ·
1901     TTATCGTGCT GAATCTGACC CTGGTGGAGA ACATCGATTT CCAGGTGATC GAGCTGTACT CTCGGGAGGA GAAGAGAATG TCTACCGTGT TCGACATCGA
         AATAGCACGA CTTAGACTGG GACCACCTCT TGTAGCTAAA GGTCCACTAG CTCGACATGA GAGCCCTCCT CTTCTCTTAC AGATGGCACA AGCTGTAGCT

         · T  M  F   R  E  Y  N   Y  Y  T   Q  R  V   T  G  L  R   R  D  L   S  D  I   A  T  N  R   N  Q  F   V  D  A
2001     GACAATGTTC CGGGAGTACA ACTACTACAC CCAGAGAGTG ACCGGCCTGA GAAGAGATCT GTCTGATATC GCCACCAACC GGAACCAGTT TGTGGATGCT
         CTGTTACAAG GCCCTCATGT TGATGATGTG GGTCTCTCAC TGGCCGGACT CTTCTCTAGA CAGACTATAG CGGTGGTTGG CCTTGGTCAA ACACCTACGA

          F  G  T  L   M  D  D   L  G  V   V  G  K  T   V  V  N   A  V  S   S  L  A  T   L  F  S   S  I  V   T  G  L
2101     TTTGGCACCC TGATGGATGA TCTGGGCGTG GTGGGCAAGA CCGTGGTGAA TGCTGTGTCC TCTCTGGCTA CCCTGTTCTC TTCTATCGTG ACCGGCCTGA
         AAACCGTGGG ACTACCTACT AGACCCGCAC CACCCGTTCT GGCACCACTT ACGACACAGG AGAGACCGAT GGGACAAGAG AAGATAGCAC TGGCCGGACT
```

```
        I N F I  K N P  F G G M  L I F  G L L  A A V V  I A V  I L L  R R R A  A S F
2201    TCAACTTCAT CAAGAATCCT TTTGGCGGCA TGCTGATCTT TGGCCTGCTG GCTGCTGTGG TGATCGCTGT GATCCTGCTG CGCAGAAGGG CTGCTTCTTT
        AGTTGAAGTA GTTCTTAGGA AAACCGCCGT ACGACTAGAA ACCGGACGAC CGACGACACC ACTAGCGACA CTAGGACGAC GCGTCTTCCC GACGAAGAAA

        ·A A N  P V Q M  I Y P  D I Q  Q I T K  Q R Q  E M N  V E P I  S K H  E L D
2301    TGCCGCTAAT CCCGTGCAGA TGATCTACCC TGATATCCAG CAGATCACCA AGCAGAGACA GGAGATGAAC GTCGAGCCTA TCTCTAAGCA CGAGCTGGAC
        ACGGCGATTA GGGCACGTCT ACTAGATGGG ACTATAGGTC GTCTAGTGGT TCGTCTCTGT CCTCTACTTG CAGCTCGGAT AGAGATTCGT GCTCGACCTG

         R I M L  A M H  D Y H  Q T K Q  D K P  D E K  E G P E  S G G  S A N  K A N
2401    AGAATCATGC TGGCCATGCA CGATTACCAC CAGACCAAGC AGGATAAGCC TGATGAGAAG GAGGGCCCTG AGTCTGGCGG CTCTGCTAAT AAGGCTAACT
        TCTTAGTACG ACCGGTACGT GCTAATGGTG GTCTGGTTCG TCCTATTCGG ACTACTCTTC CTCCCGGGAC TCAGACCGCC GAGACGATTA TTCCGATTGA

        W L N K  A K N  V L R R  R A G  Y Q P  L K R S  D S T  E S A  A A L
2501    GGCTGAACAA GGCCAAGAAC GTCCTGAGAA GAAGGGCAGG CTACCAGCCT CTGAAGAGGT CTGATTCTAC CGAGTCTGCT GCTGCCCTG
        CCGACTTGTT CCGGTTCTTG CAGGACTCTT CTTCCCGTCC GATGGTCGGA GACTTCTCCA GACTAAGATG GCTCAGACGA CGACGGGAC
```
