# Supplementary material for: Replacement of Glycoprotein B in Alcelaphine Herpesvirus 1 by Its Ovine Herpesvirus 2 Homolog : Implications in Vaccine Development for Sheep-Associated Malignant Catarrhal Fever
Source: mSphere. 2016 Aug 3;1(4):e00108-16. doi: 10.1128/mSphere.00108-16 (PMC4973634; doi:10.1128/mSphere.00108-16)
Supplement: Figure S2 [file sph004162124sf2.pdf]

A

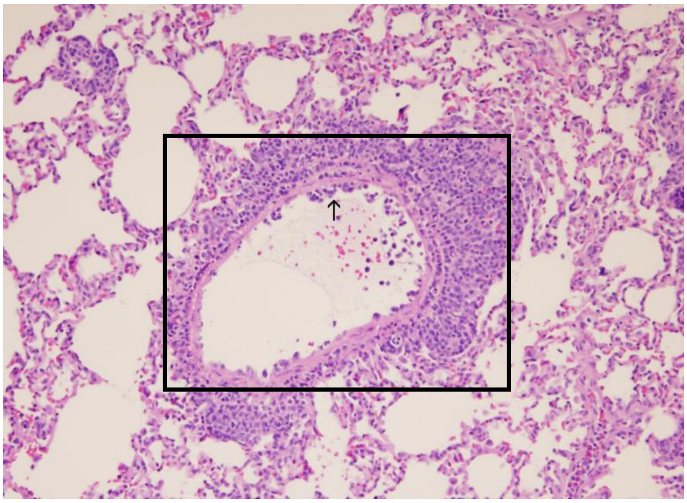

B

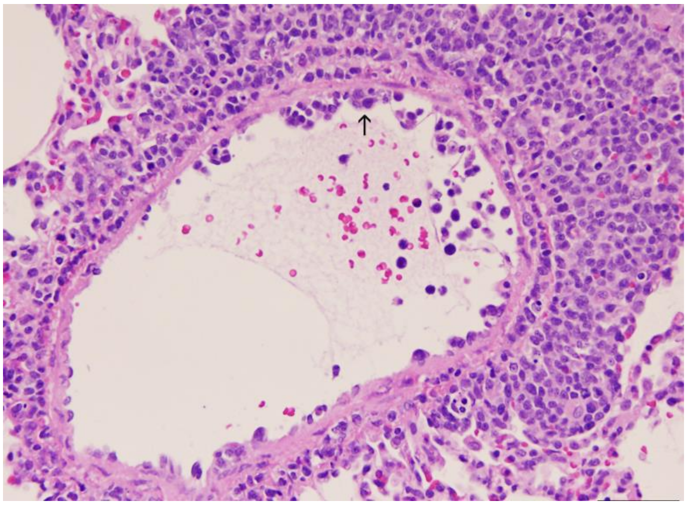

Fig. S2. Typical AlHV-1-induced lymphoproliferation in lung, associated with a vein exhibiting endophlebitis (arrow). This histopathological lesion is representative of all rabbits infected with the wild-type AlHV-1. Image A was taken at 100X. Image B is a magnification of the squared area of image A.
